# Gut microbiota develop towards an adult profile in a sex-specific manner during puberty

**DOI:** 10.1038/s41598-021-02375-z

**Published:** 2021-12-02

**Authors:** Katri Korpela, Sampo Kallio, Anne Salonen, Matti Hero, Anna Kaarina Kukkonen, Päivi J. Miettinen, Erkki Savilahti, Ella Kohva, Laura Kariola, Maria Suutela, Annika Tarkkanen, Willem M. de Vos, Taneli Raivio, Mikael Kuitunen

**Affiliations:** 1 Human Microbiome Research Program, Faculty of Medicine, University of Helsinki, Haartmaninkatu 3, P.O. Box 21, 00014 Helsinki, Finland; 2 New Childrenʼs Hospital, Pediatric Research Center, University of Helsinki and Helsinki University Hospital, Stenbäckinkatu 9, P.O. Box 347, 00029 Helsinki, Finland; 3 Laboratory of Microbiology, Wageningen University, Stippeneng 4, P.O. Box 8033, 6700 EH Wageningen, The Netherlands; 4 Translational Stem Cell Biology and Metabolism Research Program, Faculty of Medicine, University of Helsinki, Yliopistonkatu 3, P.O. Box 4, 00014 Helsinki, Finland

**Keywords:** Endocrinology, Applied microbiology

## Abstract

Accumulating evidence indicates that gut microbiota may regulate sex-hormone levels in the host, with effects on reproductive health. Very little is known about the development of intestinal microbiota during puberty in humans. To assess the connection between pubertal timing and fecal microbiota, and to assess how fecal microbiota develop during puberty in comparison with adult microbiota, we utilized a Finnish allergy-prevention-trial cohort (Flora). Data collected at 13-year follow-up were compared with adult data from a different Finnish cohort. Among the 13-year-old participants we collected questionnaire information, growth data from school-health-system records and fecal samples from 148 participants. Reference adult fecal samples were received from the Health and Early Life Microbiota (HELMi) cohort (n = 840). Fecal microbiota were analyzed using 16S rRNA gene amplicon sequencing; the data were correlated with pubertal timing and compared with data on adult microbiota. Probiotic intervention in the allergy-prevention-trial cohort was considered as a confounding factor only. The main outcome was composition of the microbiota in relation to pubertal timing (time to/from peak growth velocity) in both sexes separately, and similarity to adult microbiota. In girls, fecal microbiota became more adult-like with pubertal progression (p = 0.009). No such development was observed in boys (p = 0.9). Both sexes showed a trend towards increasing relative abundance of estrogen-metabolizing Clostridia and decreasing Bacteroidia with pubertal development, but this was statistically significant in girls only (p = 0.03). In girls, pubertal timing was associated positively with exposure to cephalosporins prior to the age of 10. Our data support the hypothesis that gut microbiota, particularly members of *Ruminococcaceae*, may affect pubertal timing, possibly via regulating host sex-hormone levels.

**Trial registration** The registration number for the allergy-prevention-trial cohort: ClinicalTrials.gov, NCT00298337, registered 1 March 2006—Retrospectively registered, https://clinicaltrials.gov/show/NCT00298337. The adult-comparison cohort (HELMi) is NCT03996304.

## Introduction

The onset of puberty is affected by genetic and environmental factors. Currently, variation in several hundred chromosomal loci are known to be associated with variation in the onset of puberty^1^, and external and internal cues such as diet^2^, exercise^3^, the amount of fat tissue^4^, intrauterine conditions^5^, psychosocial stress^6^, and chronic diseases^7^ are known to play a role.

There is rapidly growing evidence pointing to the role of gut microbiota in the regulation of growth via hormonal effects on bone growth^8^, widespread metabolic effects^9^ and effects on nutritional status^10^. Infant gut microbiota predict body mass index (BMI) at preschool age, and more specifically, *Streptococcus* and *Bacteroides* spp. are positively and bifidobacteria negatively connected to later BMI^11^. Modulation of gut microbiota in order to improve growth is routine practice in animal husbandry^12^. Gut microbiota may thus influence pubertal development via metabolic effects.

Gut microbiota are important regulators of circulating estrogens. Conjugated estrogen is secreted to bile, and certain gut microbes, particularly *Ruminococcus* and *Faecalibacterium* spp. secrete beta-glucuronidase, which deconjugates estrogen back to an active form^13^. Through the enterohepatic circulation, the deconjugated estrogens return to the systemic circulation. Several human studies have shown associations between the composition of gut microbiota and urinary and fecal estrogen levels^14,15^. In monkeys, a decrease of estrogen-metabolizing bacteria leads to a decrease in circulating estrogens^16^. In addition, dietary fiber consumption, which regulates the composition of the gut microbiota, affects the levels of serum estrogens^17–19^, suggesting an effect of gut microbiota on sex-hormone levels.

Sex hormones in connection with the composition of gut microbiota have been studied most thoroughly in mice, where gonadectomy changes the composition of the microbiota^20^, indicating an effect of sex hormones. Furthermore, it has been shown that the gut microbiota of mice influence sex-hormone levels, which in turn affects the hosts’ immune development^21,22^. In mice, gut microbiota undergo sex-specific changes during puberty that influence hormone levels^22^. Furthermore, fecal microbiota transplants (FMTs) from healthy female rats to those with polycystic ovary syndrome (PCOS) have been shown to ameliorate the condition^23^, indicating that the gut microbiota have a decisive impact on gonadal functioning in the host.

Accumulating evidence thus suggests that gut microbiota may have an important role in determining the timing of puberty via metabolic and hormonal effects. It is also possible that gut microbiota composition is dependent on hormonal signals. It has been reported that girls with idiopathic central precocious puberty (ICPP) have different gut microbiota compared with controls, especially as regards *Ruminococcus* and *Gemmiger* spp., which were found to be enriched in the ICPP group. The microbiota of ICPP patients were also more generally diverse and showed features that have been previously associated with the microbiota of obese individuals^24^.

Limited data exist on human adolescent gut microbiota. In a recent cross-sectional investigation it was discovered that the distinction in gut microbiota between sexes becomes more evident in puberty^25^. Differences have been found between adolescent and adult microbiota, and the amounts of bifidobacteria in particular have been found to diminish with age in several studies^26–29^, and age-related associations with Bacteroidetes and Firmicutes have also been reported^27–29^. However, significant individual variation exists in pubertal timing, and previous studies have not addressed the associations between pubertal stage and gut microbiota. Hence, we here investigated the association between intestinal microbiota and pubertal timing in humans in a well-characterized and longitudinally monitored cohort.

## Methods

### Participants and data sources

The study was implemented among an allergy-prevention-trial cohort including 1018 mother–child pairs where the child had a high risk of allergy^30^ (trial registration number NCT 00298337). The subjects randomly received a mixture of four probiotics in capsules: *Lactobacillus rhamnosus* GG(ATCC 53103), 5 × 10^9^ colony-forming units (cfu); *L. rhamnosus* LC705(DSM 7061), 5 × 10^9 cfu; *Bifidobacterium breve* Bb99(DSM 13692), 2 × 10^8^ cfu; and *Propionibacterium freudenreichii* ssp. shermanii JS(DSM 7076), 2 × 10^9^ cfu, or placebo. Starting 2–4 weeks before the end of pregnancy, mothers took capsules twice daily and children from day 1–2, one daily capsule and a prebiotic oligosaccharide, or placebo for the first six months of life. The treatment had no effect on growth^31^.

At the age of 13 years, 960 participants were invited for a follow-up visit. For the visit, 642 participants filled in questionnaires with information on gastrointestinal symptoms and 422 participants provided a fecal sample. Growth data on 306 participants was obtained from school health-service records^32^. Data on lifetime antibiotic use were obtained from the drug-purchase register of the Finnish National Health Insurance scheme (Kela) for all participants (Suppl. Figure 1). Written informed consent was obtained from all subjects’ parents and/or legal guardians. The study was approved by the Helsinki University Hospital Ethics Committee. The ethical statement number is 78/13/03/03/2013.

Both growth data and fecal samples were available for 65 boys and 83 girls (total 148). This sub-set was used for the majority of the analyses. Gastrointestinal symptom data were missing for 4 boys and 2 girls and antibiotic purchase data were missing for 3 girls, so the complete data was available for 61 boys and 78 girls (total 139).

Fecal samples from adults of reproductive age were obtained from Finnish Health and Early Life Microbiota (HELMi) cohort^33^, and used as reference material. A total of 396 samples from women and 444 samples from men were included. The adult reference cohort consisted of generally healthy parents of newborn infants.

### Determination of the timing of puberty from growth data

Reference growth-velocity data on prepubertal growth was obtained from previous work^34^. In total, 1127 pubertal (over eight years for girls and over 9 years for boys) measurements were available and the mean number of measurements per person was 7.6. Age at take-off of pubertal growth acceleration was defined as the age when growth velocity exceeded the age- and sex-specific mean plus 2 SD for the first time, followed by a period of accelerated growth typical for puberty. Age at peak-height velocity (APHV) was defined as the age when a participant’s growth velocity was highest during a phase of accelerated growth after eight and nine years of age in girls and boys, respectively. Only measurements that were at least six months apart were used for growth-velocity calculation.

For 11 boys and 23 girls, it was only possible to determine APHV according to the aforementioned criteria. In these cases, the age at take-off was imputed to be six months prior to APHV.

To assess the correlation between gut microbiota and the timing of puberty, two new variables were derived. First, the time between growth take-off and sample collection, and second, the time between APHV and sample collection.

### Fecal sample analysis

Fecal samples were collected by the participants at home and frozen immediately. They were transported to the laboratory frozen and stored at − 80 °C until processing.

Bacterial DNA was extracted using a previously described repeated bead-beating method^35^ with the following modifications for automated DNA purification: ca. 125 mg of fecal material were suspended in 1 ml of sterile ice-cold PBS, and 175 μl of fecal suspension was combined with 235 μl of RBB lysis buffer (500 mM NaCl, 50 mM Tris–HCl (pH 8.0), 50 mM EDTA, 4% SDS) in a bead-beating tube from the Ambion Magmax™ Total Nucleic Acid Isolation Kit (Life Technologies, Carlsbad, CA, USA). After repeated bead-beating, 200 μl of the supernatant was used for DNA extraction with a KingFisher™ Flex automated purification system (ThermoFisher Scientific, Waltham, MA, USA) using a MagMAX™ Pathogen High Vol. Duo program. DNA was quantified using a Quanti-iT™ Pico Green dsDNA assay (Invitrogen, San Diego, CA, USA) and 1 ng was used for V3–V4-region amplicon PCR of the 16S rRNA gene as previously described^36^. Sequencing was carried out with Illumina HiSeq 2500 equipment in Rapid Run mode.

### Statistical analysis and visualization

The sequencing reads were processed using the ProcessReads and TaxonomicTables functions in the R package mare^37^. The forward reads were quality and chimera filtered using USEARCH^38^ v 8.1 and mapped to the Silva^39^ reference database. After mapping the reads to the reference database, the reads were summarized at different taxonomic levels.

Statistical analysis was conducted in R^40^, partly using the R package mare, which relies on the R packages vegan^41^, nlme^42^, and MASS^43^. Plots and other figures were made with R^40^ using the R package ggplot2^44^ and Microsoft Powerpoint for Mac^45^.

Similarity to microbiota in adults was assessed as the correlation between the log-transformed relative abundances of the bacterial taxa at species level between each participant and an adult of the same sex. The average similarity to adults for each participant was taken as a measure of microbiota maturity.

Associations between bacterial taxa and pubertal timing were analyzed using regression as described below. The number of reads per taxon was used as the response variable and the model was adjusted for probiotic use, time since the last antibiotic course, BMI and whole-grain intake as a proxy of dietary-fiber intake. The total number of reads per sample was used as an offset. For each taxon, a suitable model was identified based on standard statistical diagnostics. Initially, a generalized linear model (glm) with negative binomial distribution was fitted. In cases in which the model did not converge, a glm with Poisson distribution was fitted, and if that did not converge, finally a linear model (lm) using relative abundances instead of raw read counts was fitted (log-transformed if necessary depending on the data distribution). In cases in which there were patterns in the residuals, a generalized least squares (gls) model was fitted, also modelling the residual variation. If no model fulfilled standard statistical diagnostic criteria (normality and stochasticity of residuals), no p-value was obtained. Using this iterative approach, a suitable statistical model was identified for each taxon.

## Results

Comparison of the whole allergy-prevention-trial-cohort at 13-years and the analysed cohort is presented in Suppl Table 1. The groups were reasonably similar, except for the prevalence of asthma (16.8% in whole cohort vs 7.2% in the analysed cohort). The difference was statistically significant (p = 0.004). The birth weight in whole cohort (average 3588 g) was higher compared to analyzed cohort (average 3452 g) and the difference was statistically significant (p = 0.002).

At 13 years of age, the boys, as expected, were less mature in terms of pubertal development than the girls (Suppl. Figure 2). Girls were mostly (81%) past their peak-growth velocity, while most boys (80%) had not yet reached it. Self-reported Tanner staging reflected a similar picture (Suppl. Table 2).

**Figure 1:**
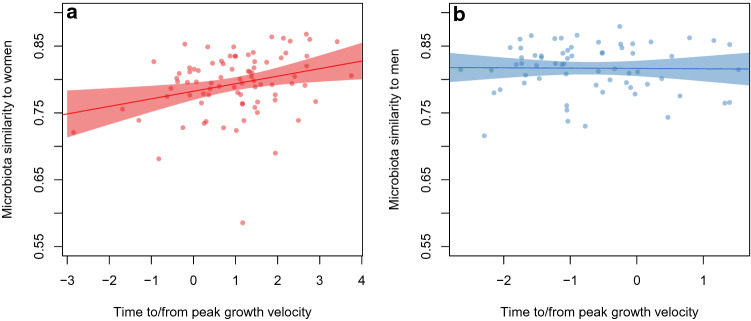
(**a**) Microbiota similarity to that in adults in relation to pubertal timing (time to/from peak growth velocity) in girls. (**b**) Microbiota similarity to that in adults in relation to pubertal timing (time to/from peak growth velocity) in boys.

Gut microbiota were analyzed in fecal samples by extracting DNA, amplifying the 16S rRNA gene and sequencing the amplicons. To compare the compositions of gut microbiota in children and adults of the same sex, a microbiota maturity index was created. In girls, similarity to adult-female microbiota was positively correlated with pubertal progress: the more progressed the girl was in terms of puberty, the more similar were the microbiota (Fig. 1a, p = 0.009). The same was not evident as regards boys versus adult males (Fig. 1b, p = 0.9). The association between the time from growth take-off and microbiota maturity was similar to that of the time from peak growth velocity to maturity of the microbiota (girls, p = 0.01; boys, p = 0.85; Suppl. Figure 3). We tested whether antibiotic exposure, BMI, intake of probiotics, and intake of whole-grain foods (based on dietary questionnaires) could explain the association between pubertal timing and the overall similarity to microbiota in adults. However, after adjustment for each of these variables, the association remained significant in girls (p < 0.05).

**Figure 2:**
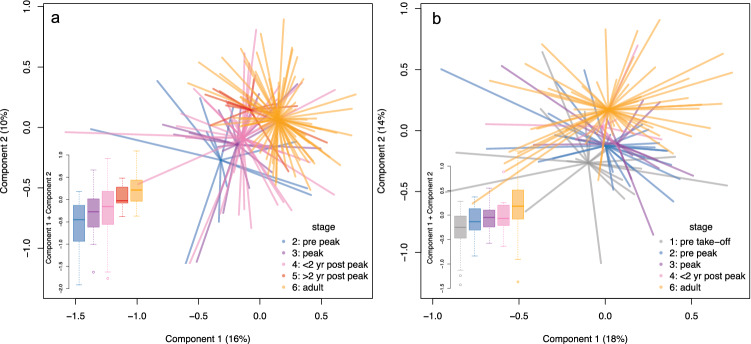
(**a**) Principal coordinates analysis of gut microbiota composition in girls compared with adults of the same sex. (**b**) Principal coordinates analysis of gut microbiota composition in boys compared with adults of the same sex.

To assess the information on growth take-off and peak growth velocity, the children were divided into groups: prior to growth take-off (N = 18/0 boys/girls), prior to peak growth (post take-off, up to three months before peak, N = 27/11 boys/girls), peak growth (three months before to six months after peak, N = 13/17 boys/girls), immediately after peak growth (six months to two years after peak, N = 8/40 boys/girls) and more than two years after peak growth (N = 0/15 boys/girls). Principal coordinates analysis revealed that these groups differed in microbiota composition in both sexes, with the composition becoming more adult-like as puberty progressed, especially in girls (Fig. 2a). In girls, the summed score of the first two principal coordinates in the two post-peak groups was significantly higher than the score in the pre-peak group (< 2 years post-peak, p = 0.02; > 2 years post-peak, p = 0.0004). In boys, the same trend was evident (Fig. 2b), although nonsignificant (compared with pre-take-off: pre-peak, p = 0.09; peak, p = 0.06; < 2 years post-peak, p = 0.07).

**Figure 3:**
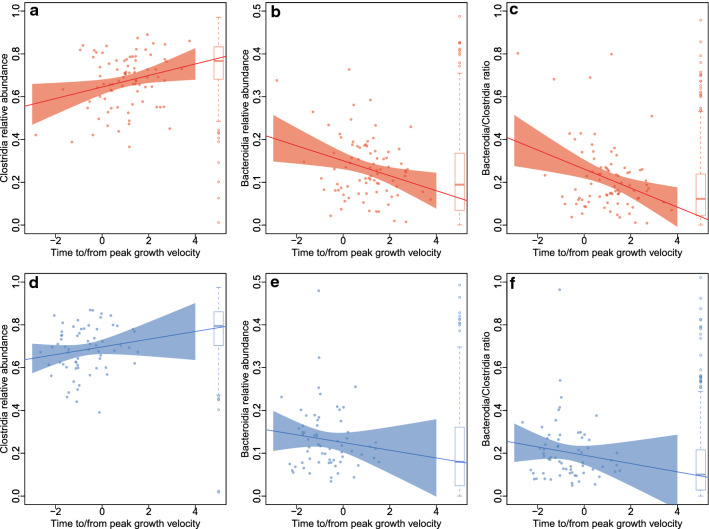
(**a**) Abundance of Clostridiales in relation to pubertal timing (time to/from peak growth velocity) in girls. (**b**) Abundance of Bacteroidales in relation to pubertal timing (time to/from peak growth velocity) in girls. (**c**) Bacteroidales/Clostridiales ratio in relation to pubertal timing (time to/from peak growth velocity) in girls. (**d**) Abundance of Clostridiales in relation to pubertal timing (time to/from peak growth velocity) in boys. (**e**) Abundance of Bacteroidales in relation to pubertal timing (time to/from peak growth velocity) in boys. (**f**) Bacteroidales/Clostridiales ratio in relation to pubertal timing (time to/from peak growth velocity) in boys.

The main driver behind maturation of the microbiota in girls was a change in the relative abundances of the two most abundant classes of bacteria, Clostridiales and Bacteroidales (Fig. 3a–c). During puberty, the relative abundance of Clostridiales increased (p = 0.03, after adjusting for BMI, whole-grain intake, probiotic use and antibiotic exposure) and the relative abundance of Bacteroidales decreased (p = 0.03) towards adult-like levels. The same trends, although nonsignificant (Clostridiales, p = 0.14; Bacteroidales, p = 0.48), were evident in boys (Fig. 3d–f). At phylum level, the abundance of the Firmicutes increased with puberty progression in both sexes, significantly in girls (p = 0.01) and nonsignificantly in boys (p = 0.23), and the abundance of Bacteroidetes decreased (girls, p = 0.03; boys, p = 0.48).

At family and genus levels, the puberty-associated changes in the microbiota of girls were mainly attributable to an increase in members of Ruminococcaceae (*Anaerofilum*, p = 0.02; *Anaerotruncus*, p < 0.0001; *Subdoligranulum*, p = 0.02; uncultured, p = 0.002) and *Lachnospiraceae* (*Dorea*, p = 0.03; *Syntrophococcus*, p = 0.005) and a decrease of the order Bacteroidales (p = 0.03), especially *Paludibacter* (p = 0.04), *Macellibacteroides* (p = 0.005) and *Barnesiella* (p < 0.001). The same patterns were evident in boys, with only *Barnesiella* (p < 0.001), *Macellibacteroides* (p < 0.001) and uncultured *Ruminococcaceae* (p = 0.02) reaching statistical significance. In addition to the abundant taxa, there was a decline in low-abundance taxa *Streptococcus* (girls, p = 0.02; boys, p = 0.6), *Lactobacillu*s (girls, p = 0.5; boys, p = 0.009), *Coriobacteriaceae* (girls, p = 0.1; boys, p = 0.04), and *Escherichia* (girls, p = 0.3; boys, p < 0.001).

**Figure 4:**
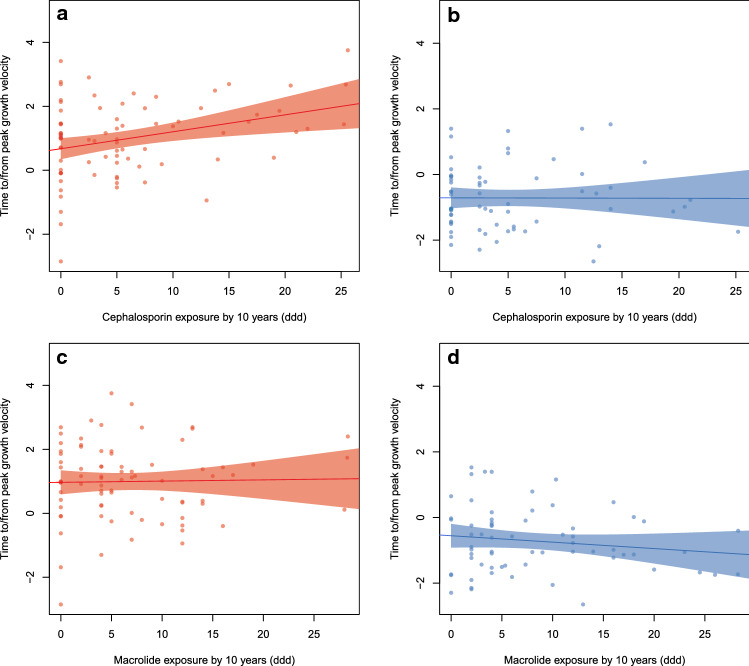
(**a**) Association between exposure to cephalosporin and pubertal timing (time to/from peak growth velocity) in girls. (**b**) Association between exposure to cephalosporin and pubertal timing (time to/from peak growth velocity) in boys. (**c**) Association between exposure to macrolide, and pubertal timing (time to/from peak growth velocity) in girls. (**d**) Association between exposure to macrolide, and pubertal timing (time to/from peak growth velocity) in boys.

We considered the possibility that antibiotic exposure might influence the timing of puberty. For each sex, we identified the antibiotic type and timing of exposure that was most strongly associated with pubertal timing, using AIC-based model selection. For girls, the most strongly associated antibiotic variable was (first- and second-generation) cephalosporin exposure (cumulative defined daily doses, ddd) up to the age of 10 years, which was positively associated with pubertal progress (p = 0.004, Fig. 4a). In boys, cephalosporin exposure was not associated with the timing of puberty (p = 0.97, Fig. 4b). For boys, the most strongly associated variable was macrolide exposure by the age of 10 years, which showed a non-significant negative trend (p = 0.12, Fig. 4d). In girls, macrolide use was not associated with puberty (p = 0.66, Fig. 4c). We also tested for an association between BMI and pubertal timing. In both sexes, there was a modest but nonsignificant, positive trend (Suppl. Figure 4).

**Figure 5:**
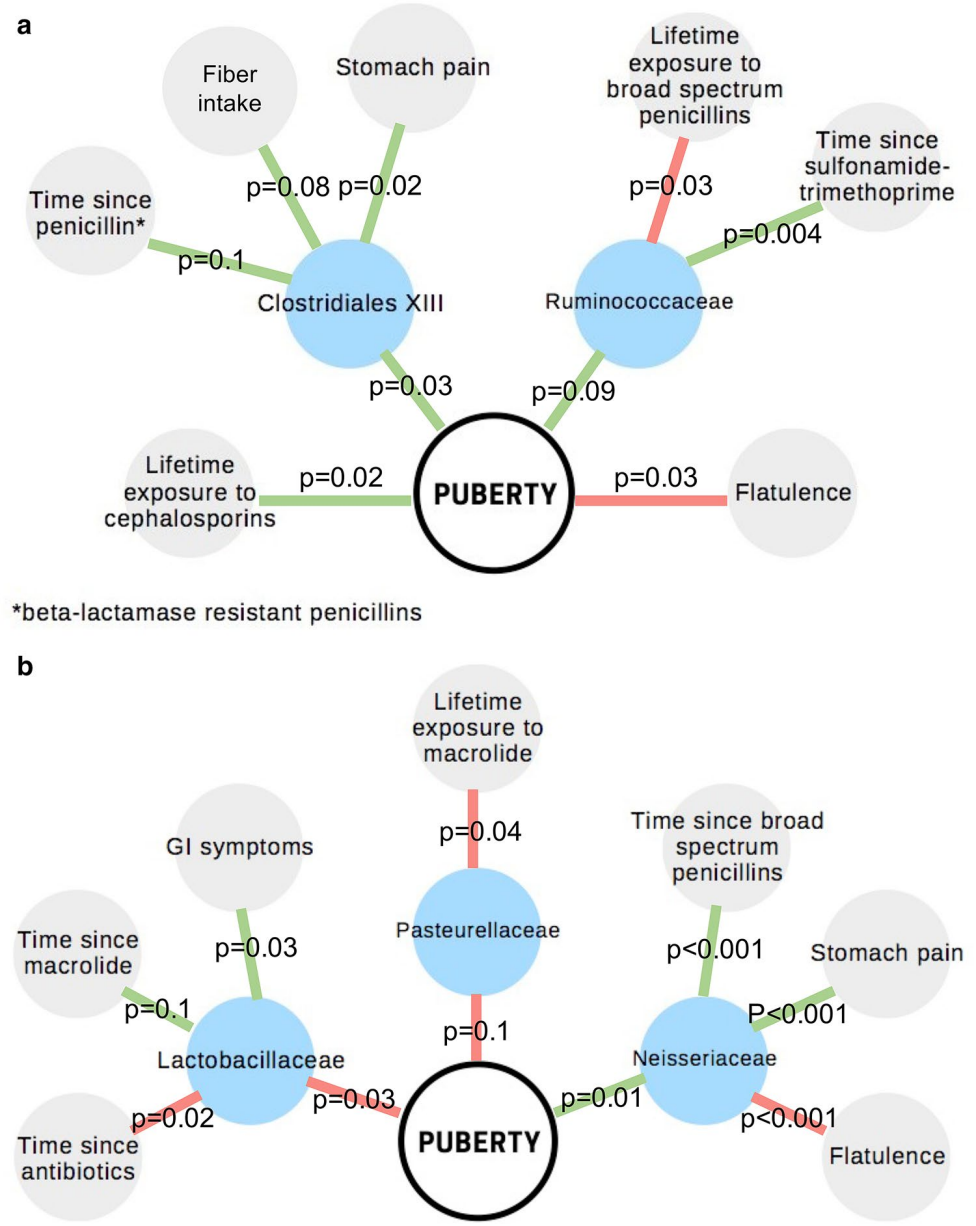
(**a**) Multivariate model for pubertal timing in girls. (**b**) Multivariate model for pubertal timing in boys.

Finally, we created multivariate models for the timing of puberty, including gut microbiota at family level, detailed information on antibiotic exposure (time since last antibiotic course per antibiotic type, cumulative lifetime number of courses and ddd per antibiotic type), probiotic use, BMI, and gastrointestinal (GI) symptoms as potential confounders and explanatory variables for both the timing of puberty and for the relative abundances of the bacterial taxa. We used AIC-based model selection to arrive at the final model. Among girls, pubertal timing was associated positively with the abundance of Clostridiales family XIII bacteria and *Ruminococcace*ae, both members of the Clostridia class (Fig. 5a). In addition, in a multivariate model, timing of puberty was associated positively with lifetime exposure to cephalosporins and negatively with self-reported flatulence. In boys, a very different picture emerged. Timing of puberty was associated negatively with *Lactobacillaceae* and *Pasteurellaceae* and positively with *Neisseriaceae* (Fig. 5b). The latter taxa were associated with antibiotic exposure and with GI symptoms. In boys, the negative association between puberty and flatulence appeared to be mediated by *Neisseriaceae*.

## Discussion

In this work we aimed to investigate the relationship between the timing of puberty and gut microbiota. Our results show, for the first time, that gut microbiota shifts towards adult-like composition as the puberty progress. These findings suggest an association between sex hormones and gut microbiota development.

Pubertal timing in girls was associated with antibiotic use, especially cephalosporin. It is already known that antibiotic exposure, very likely via changes in the gut microbiota, is connected to higher BMI^46,47^, and higher BMI is connected to early puberty^48^. However, in our dataset BMI was not significantly associated with pubertal timing and the association with cephalosporin use was also observable when adjusting for BMI as a confounding factor. This suggests that antibiotic-driven changes in gut microbiota may be causally related to pubertal timing, independently of the metabolic effects. However, the connection was not visible in boys in our cohort.

Comparison of pubertal and adult microbiota revealed that during puberty, girls’ gut microbiota develops in the direction of adult women’s microbiota, and the level of similarity is related to the degree of pubertal development. Importantly, the trend towards adult-like microbiota was visible in the first two principal coordinates, indicating that the main dimensions of variation in the gut microbiota are affected by pubertal progression in this age group. Among boys a similar trend was observed with respect to the first two principal coordinates (together explaining 32% of total microbiota variation), but not when considering the whole microbiota composition. It is possible that the route of microbiota development is different in men compared with women, but the most probable explanation for the difference is the lack of data from boys past the period of peak growth velocity, as a result of the generally later onset of puberty in boys, resulting in too few cases in this time window.

For both sexes, the dominant taxa Clostridia, including the abundant families *Ruminococcaceae* and *Lachnospiraceae*, and Bacteroidia, including the abundant genus *Bacteroides*, and their ratio, approached adult levels in parallel with pubertal development. The result shows that development of the gut microbiota that begins at the period of weaning^49^, with increasing abundance of Clostridia, still continues through puberty. Our data show that children do not achieve an adult-like gut microbiota composition before the later stages of puberty.

The increasing relative abundance of Clostridia with pubertal development is supported by earlier findings. An Italian study revealed a lower abundance of Bacteroidetes and a higher abundance of Firmicutes in normal-weight adults compared with normal-weight adolescents, but the pattern was opposite in obese individuals ^29^. In contrast, American study reported a higher abundance of Bacteroidetes in adults compared with 7- to 12-year-olds ^28^, corresponding to the pattern seen in obese Italians. The weight status of the participants in the American study was not reported. A Dutch study in which 6- to 9-year-old children were compared with adults showed a lower abundance of *Bacteroides* and higher abundances of *Ruminococcus*, *Eubacterium* and *Clostridium* species in adults^50^. We had mostly normal-weight individuals, with only four obese girls and five obese boys. Owing to small numbers it was not possible to reliably address whether or not gut microbiota development was different between obese and normal-weight adolescents, but adjusting for BMI in the models did not alter the results. It is possible that obesity or, for example, a high-fat diet could alter microbiota progression during puberty. In contrast to our findings, a recent Chinese study showed a higher abundance of Clostridia in 5-year-olds compared with 15-year-olds^51^. In the 15-year-olds, several genera were found to be associated with the level of serum testosterone, but estradiol concentrations were not associated with microbiota composition^51^.

Previous studies on the association between gut microbiota and reproductive stage in humans have shown that at the end of female reproductive life, the pattern that we observed reverses again, with increasing abundance of Bacteroidetes and decreasing abundances of Firmicutes during menopause^52^. Together, these results strongly indicate that sex hormones are a major driver of gut microbiota composition, particularly affecting the balance between the two major phyla, Firmicutes and Bacteroidetes. Furthermore, *Bacteroides* spp. were positively and *Ruminococcus* spp. negatively correlated with testosterone levels in a cohort of women with and without polycystic ovary syndrome^53^. Several *Ruminococcaceae* OTUs (operational taxonomic units) were at a lower abundance in women with polycystic ovary syndrome^53^, suggesting that their abundance is dependent on normal gonadal function of the host.

Several types of Clostridia, especially species of the *Ruminococcaceae* genera *Faecalibacterium* and *Ruminococcus*, are known to reactivate inactive conjugated forms of estrogen via their beta-glucuronidase activity^13^. The beta-glucuronidase enzymes of *Ruminococcus* and *Faecalibacterium* spp. have the ability to cleave both estrone and estradiol, while the enzyme of *Bacteroides fragilis* shows catalytic activity only to estrone—orders of magnitude weaker than that of the Clostridial enzymes^13^. The relative abundance levels of members of the *Ruminococcaceae* family have been positively correlated with levels of circulating estrogens^14^. In accordance with the higher estrogen-metabolizing activity of *Ruminococcus* spp., the ratio of estrogen metabolites to estrogen parent compounds in urine has been shown to correlate positively with the relative abundance of *Ruminococcus* and negatively with that of *Bacteroides* spp.^15^. Based on these data and our current results, it is plausible that gut microbiota may partly regulate the onset of puberty and menopause via their estrogen metabolism. This hypothesis suggests that microbiota-targeting treatments may be effective in regulating estrogen levels. Indeed, increased consumption of dietary fiber reduces the levels of circulating estrogens^18^. The effect has been shown to be caused specifically by wheat bran, but not by oat or corn bran^19^. Notably, wheat-bran consumption decreases the relative abundance of *Ruminococcaceae*, which are specialized in resistant starch fermentation^17^.

In addition to their estrogen metabolism, Clostridia and Bacteroidia, the two dominant bacterial classes in the adult human gut, have widespread and varying effects on host physiology^9^. The families *Lachnospiraceae* and *Ruminococcaceae* contain the most important butyrate producers, which are adapted to fermenting complex plant-based carbohydrates and are generally considered important for a healthy gut^9^. Members of the *Ruminococcaceae* family have been associated with healthy growth in a cohort of undernourished young children^10^.

While the capacity of specific gut microbes to metabolize estrogen suggests that gut microbiota may regulate puberty, the reverse is also possible. Sex hormones might affect maturation of the microbiota by directly affecting the growth of specific taxa, by influencing immune reactions to gut microbes, by affecting bile-acid composition^20^, or in some as yet uncharacterized manner. More investigation is warranted to better understand the underlining mechanisms.

There are some limitations in our study. Our investigation did not include hormone level measurements, and so the pubertal timing is based on the changes in growth velocity only. For boys 13 years-of-age was probably not the optimal time point to investigate puberty-related changes in gut microbiota, likely resulting in weaker results than in the girls.

The sample size in this study was substantially smaller than the whole 13-year follow-up sample. This was due to lack of growth and microbial data. The main reason for the limited availability of the growth data is, that some of the participants have moved away from the capital-city region of Finland, and therefore we couldn’t attain the growth information from the school health-service records of the local municipalities. In addition, many participants were unwilling to provide faecal samples, which contributed to the reduced sample size. However, it is unlikely that willingness to provide a faecal sample or continued residence in the capital region would confound the association between puberty and gut microbiota.

Our study population was selected on the basis of its high risk of allergic disease—they have at least one parent with atopic disease. The population was collected from a relatively limited geographic area in the capital-city region of Finland (population 1.5 million inhabitants and population density 800–3000 inhabitants/km^2^), 50% living in urban, 40% suburban and 10% in rural areas. These factors may affect the generalization of our findings.

## Conclusion

Previously very little was known about the development of human gut microbiota in puberty. Our investigation opens interesting insights to the connection of the gut microbiota and pubertal development. Our data shows, that the development is sex-specific. Gut microbiota were connected to pubertal timing on girls, but not on boys, possible due to later development pattern on boys. The timing of puberty may be affected by the gut microbiota, particularly members of *Ruminococcaceae*, which may affect the timing via regulating host sex-hormone levels. Although we didn’t have hormonal data, growth curve analysis is a valid method to estimate the timing of puberty, even in the absence of serial measurements of gonadotropin and sex steroid levels. Pubertal timing on girls correlates with prepubertal antibiotic usage. The effect of lifestyle factors on pubertal timing may be mediated via changes in gut microbiota. More investigation is warranted to evaluate the generalizability of the findings and to clarify the cause-and-effect relations.

### Ethics approval and consent to participate

All experiments were performed in accordance with relevant guidelines and regulations. Written informed consent was obtained from all subjects’ parents and/or legal guardians. The study was approved by the Helsinki University Hospital Ethics Committee. The ethical statement number is 78/13/03/03/2013.

## Supplementary Information

Supplementary Figure legends.

Supplementary Figure 1.

Supplementary Figure 2.

Supplementary Figure 3.

Supplementary Figure 4.

Supplementary Table 1.

Supplementary Table 2.

## Data Availability

The raw dataset analysed during the current study is available in the European Nucleotide Archive (ENA) at EMBL-EBI under accession number PRJEB44673 (https://www.ebi.ac.uk/ena/browser/view/PRJEB44673).
