# Supplementary material for: Gut microbiota develop towards an adult profile in a sex-specific manner during puberty
Source: Sci Rep. 2021 Dec 2;11:23297. doi: 10.1038/s41598-021-02375-z (PMC8640005; doi:10.1038/s41598-021-02375-z)
Supplement: Supplementary file 1 — Supplementary Figure legends. [file 41598_2021_2375_MOESM1_ESM.pdf]

Suppl. Fig. legends

Suppl. Fig. 1 Selection of the study population on the basis of availability

Suppl. Fig. 2 Distribution of pubertal timing (time to/from peak growth velocity)

Suppl. Fig. 3 Microbiota similarity to that in adults in relation to pubertal timing (time to/from growth take-off)

Suppl. Fig. 4 Association between BMI and puberty timing (time to/from peak growth velocity)
