# Supplementary figures and images for: Gut microbiota develop towards an adult profile in a sex-specific manner during puberty
Source: Sci Rep. 2021 Dec 2;11:23297. doi: 10.1038/s41598-021-02375-z (PMC8640005; doi:10.1038/s41598-021-02375-z)

Suppl. Fig. 1

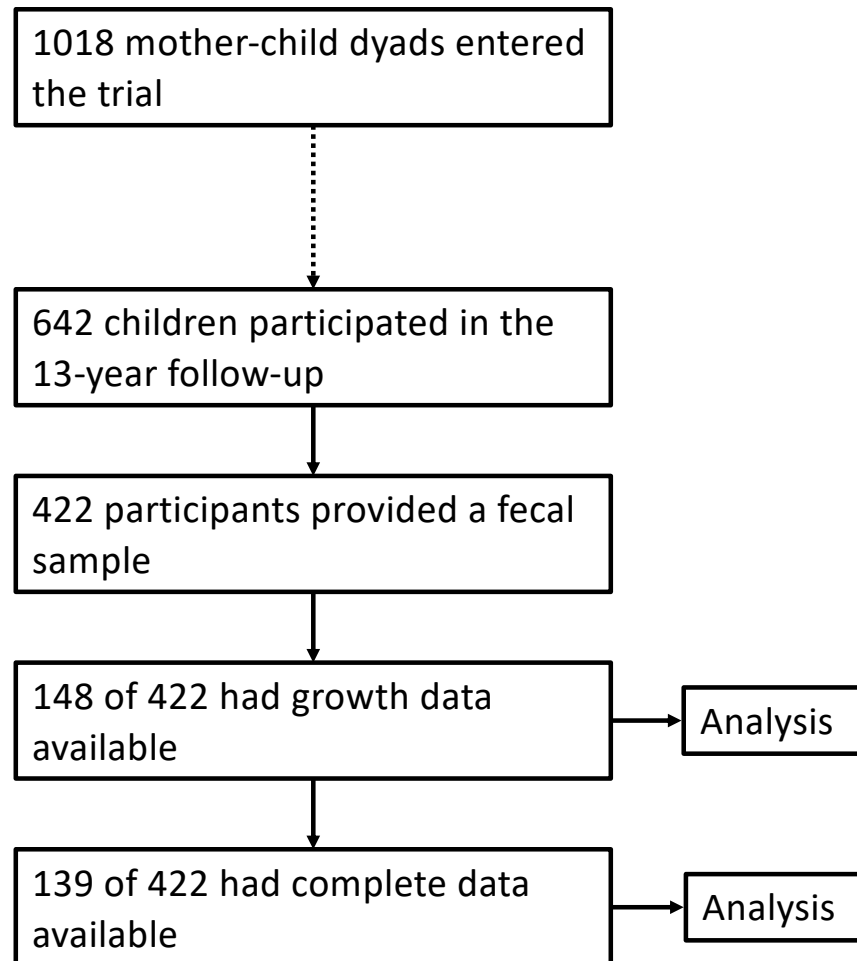

Supplement: Supplementary file 2 — Supplementary Figure 1. [file 41598_2021_2375_MOESM2_ESM.pdf]

Suppl. Fig. 2

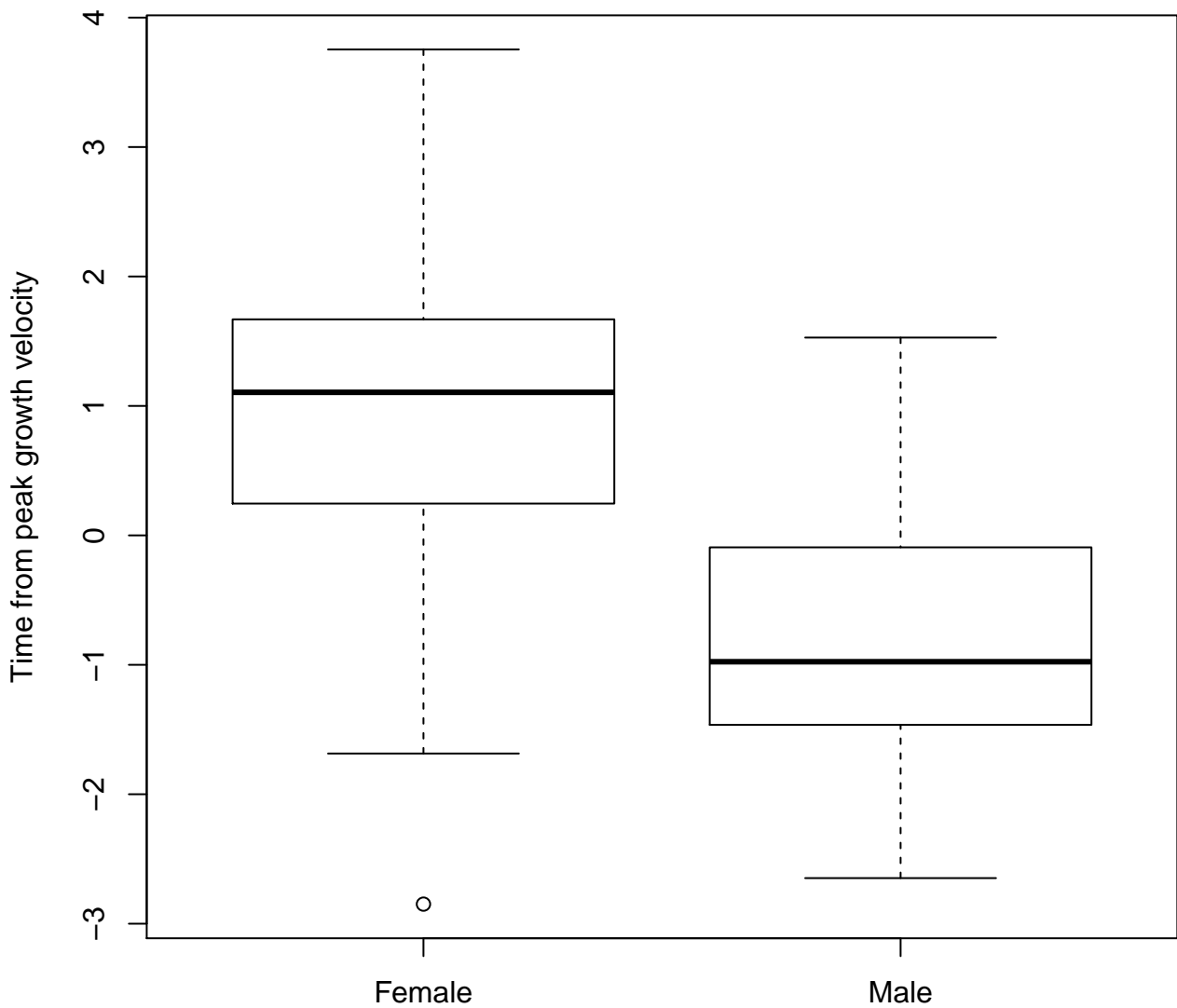

Supplement: Supplementary file 3 — Supplementary Figure 2. [file 41598_2021_2375_MOESM3_ESM.pdf]

Suppl. Fig. 3

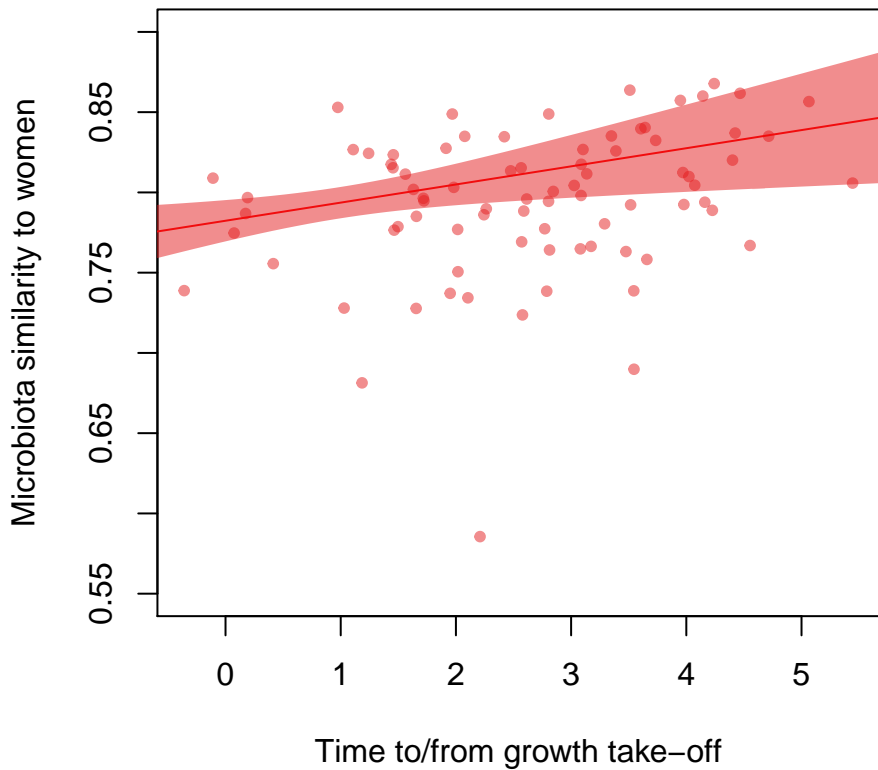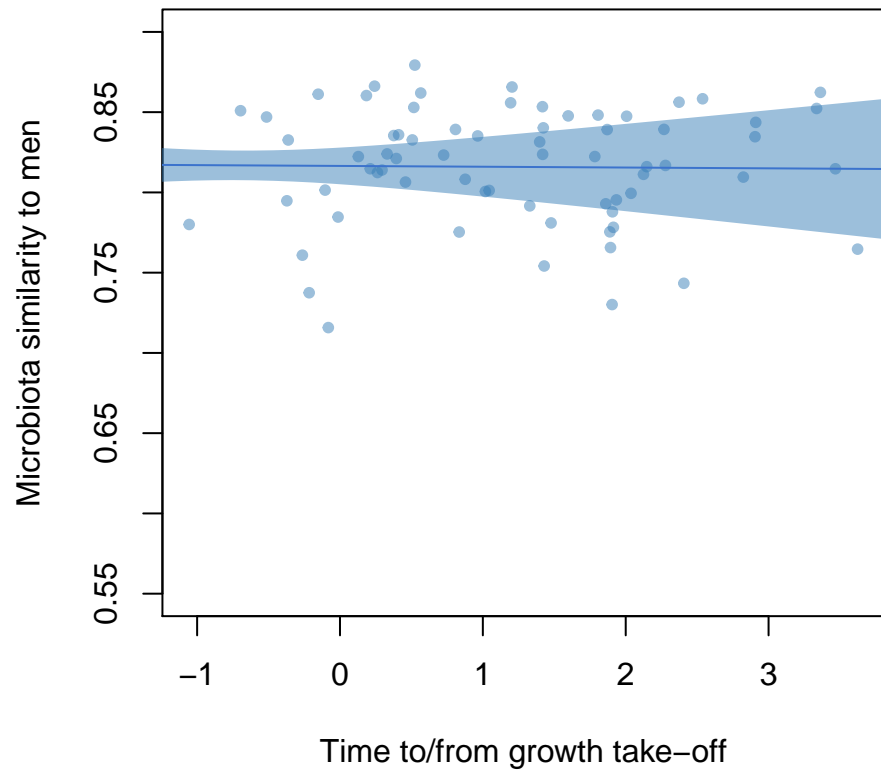

Supplement: Supplementary file 4 — Supplementary Figure 3. [file 41598_2021_2375_MOESM4_ESM.pdf]

Suppl. Fig. 4

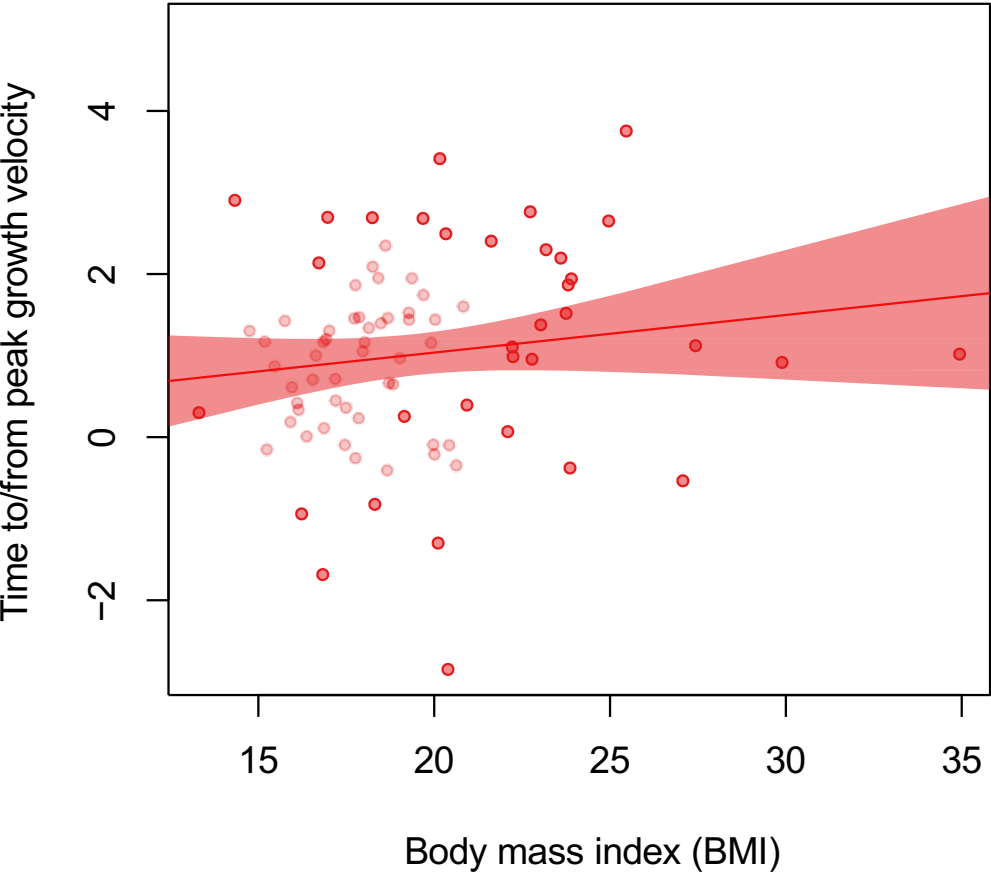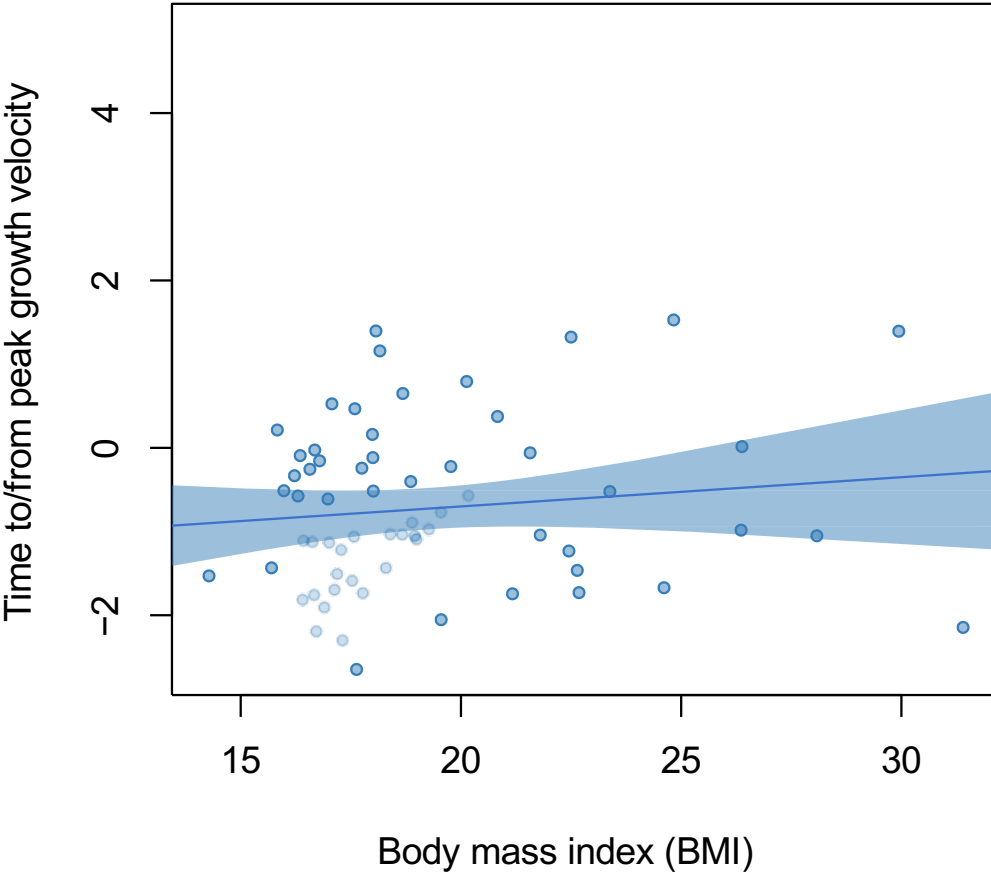

Supplement: Supplementary file 5 — Supplementary Figure 4. [file 41598_2021_2375_MOESM5_ESM.pdf]
