# Supplementary material for: Gut microbiota develop towards an adult profile in a sex-specific manner during puberty
Source: Sci Rep. 2021 Dec 2;11:23297. doi: 10.1038/s41598-021-02375-z (PMC8640005; doi:10.1038/s41598-021-02375-z)
Supplement: Supplementary file 6 — Supplementary Table 1. [file 41598_2021_2375_MOESM6_ESM.pdf]

Suppl. Table 1
Comparison of the whole cohort participating 13-year follow-up and the analyzed cohort

	Whole cohort (n=642)			Analyzed cohort (n=139)		
	All	Boys	Girls	All	Boys	Girls
Girls	50.2% (322)	-	-	56.1% (78)	-	-
Birth weight (g) (SD)	3588 (484)	3498 (458)	3678 (493)	3452 (468)	3429 (409)	3481 (537)
Birth height (cm) (SD)	50.6 (2.0)	50.0 (1.9)	51.0 (2.0)	50.2 (2.2)	49.8 (1.8)	50.7 (2.5)
Born with caesarean section	17.0% (109)	17.1% (55)	16.9% (54)	15.8% (22)	12.8% (10)	19.7% (12)
Parents with higher education	51.9% (333)	49.1% (158)	54.7% (175)	56.8% (79)	56.4% (44)	57.4% (35)
Asthma	16.8% (108)	13.4% (43)	20.3% (65)	7.2% (10)	5.1% (4)	9.8% (6)
Other chronic disease	11.7% (75)	11.5% (37)	11.9% (38)	13.7% (19)	11.5% (9)	16.4% (10)
BMI at 5 years (SD)	15.8 (1.3)	15.8 (1.5)	15.8 (1.2)	15.6 (1.4)	15.6 (1.5)	15.7 (1.3)
Uses fiber rich foods daily	71.2% (457)	71.1% (229)	71.2% (228)	62.6% (87)	61.5% (48)	63.9% (39)
