# Supplementary material for: Gut microbiota develop towards an adult profile in a sex-specific manner during puberty
Source: Sci Rep. 2021 Dec 2;11:23297. doi: 10.1038/s41598-021-02375-z (PMC8640005; doi:10.1038/s41598-021-02375-z)
Supplement: Supplementary file 7 — Supplementary Table 2. [file 41598_2021_2375_MOESM7_ESM.pdf]

Suppl. Table 2
Self-reported Tanner staging (pubic hair) of the participants

	Boys	Girls
Stage		
1	26.2% (16)	14.8% (12)
2	32.8% (20)	27.2% (22)
3	23.0% (14)	46.9% (38)
4	16.4% (10)	6.2% (5)
5	1.6% (1)	4.9% (4)
